# Evaluation of Lung Contusion, Associated Injuries, and Outcome in a Major Trauma Center in Shiraz, Southern Iran

**DOI:** 10.1155/2021/3789132

**Published:** 2021-04-22

**Authors:** Parviz Mardani, Mohammad Moayedi Rad, Shahram Paydar, Armin Amirian, Reza Shahriarirad, Amirhossein Erfani, Keivan Ranjbar

**Affiliations:** ^1^Thoracic and Vascular Surgery Research Center, Shiraz University of Medical Science, Shiraz, Iran; ^2^Department of Surgery, Shiraz University of Medical Science, Shiraz, Iran; ^3^Trauma Research Center, Shiraz University of Medical Science, Shiraz, Iran; ^4^Student Research Committee, Shiraz University of Medical Science, Shiraz, Iran

## Abstract

**Objective:**

Blunt chest trauma as one of the most common injuries in trauma cases can cause significant morbidity and mortality. The purpose of this study was to determine the clinical course of traumatic injuries with an initial diagnosis of a pulmonary contusion in patients.

**Method:**

In this retrospective study, we evaluated the demographic and clinical features of patients who were referred to a major trauma center in southern Iran. In our study, patients were enrolled with the diagnosis of pulmonary contusion. All included patients were above 16 years of age, with an initial CT scan in favor of pulmonary contusion, while patients not being hospitalized for more than 48 hours were excluded from the study.

**Results:**

Among the 434 patients included in our study, 366 (84%) were male and the mean age was 41.17 (SD = 17.89). Among them, the majority (80.4%) had right side lung contusion and 47 patients (10.8%) had right rib fracture. The most common injuries were head and neck injury (56.9%) and limbs (30%). In 25% of cases, pulmonary contusion was associated with pneumothorax and 15.8% with hemothorax. Also, 49.6% of patients were transferred to the ICU. The mortality rate in our study was 15.2% (*n* = 66).

**Conclusion:**

Although recent advances in pulmonary care and ventilator management have been achieved, there is still considerable morbidity and mortality associated with this condition. Therefore, there is a need to provide a national guideline based on native patient information for better management.

## 1. Introduction

Blunt chest trauma is considered as one of the most common problems of health practice, especially with the increase of road traffic accidents accounting for almost 15% of trauma cases [1]. Although the majority of thoracic bony structure fractures are nonthreatening and can be managed with no hospital admission, chest injury itself can be the primary cause of death in patients with multiple trauma and its complications responsible for more than 25% of deaths in patients with blunt trauma [2]. On average, one-third of patients with thoracic trauma develop rib fractures, which increases the risk of death and disability as the number of fractured ribs increases [3]. Moreover, complications such as pneumonia, sepsis, multiorgan failure, hemorrhagic shock, and pneumomediastinum (caused by the Macklin effect) have been reported in other studies [4, 5].

Pulmonary contusion, which is one of the most frequent injuries in the context of blunt trauma, has a prevalence of 25 to 75% in various studies. Pulmonary contusion refers to the damage to the alveolar vessels without pulmonary tissue laceration, which deteriorates the patients slowly within the first 24 to 48 hours and results in the leakage of blood and fluid into the alveolar space adjacent to the damaged parenchyma. Furthermore, the subsequent edema increases the damage done to the lung structure, which results in the disruption of gas exchange, perfusion, and ventilation [6, 7].

It has been reported that 85 percent of severe pulmonary contusions requiring mechanical ventilation were associated with severe lateral chest wall injury. Pulmonary contusion is a risk factor for ARDS, pneumonia, long-term respiratory failure, and death [7–12]. Thus, considering the diagnosis of pulmonary contusion is essential as many cases are characterized by chest computed tomography (CT) alone [13].

Pulmonary contusion is also commonly reported to occur in severe injuries with Injury Severity Score (ISS) above 15. Some patients admitted to the intensive care unit (ICU) may require invasive mechanical ventilator support if pulmonary contusion is associated with severe chest trauma accompanied by severe hypoxia. Intubation rates vary between 23% and 75% among patients with thoracic trauma and are most strongly associated with the severity of trauma and subsequent injuries such as hemorrhagic shock, brain injury, and underlying brain disease. It has been reported that invasive mechanical ventilation can be able to improve oxygenation but can also cause ventilator-related lung damage [14].

## 2. Material and Method

We retrospectively evaluated all trauma patient records admitted to Shahid Rajaee Hospital (affiliated by the Shiraz University of Medical Science, verified as a major trauma center) cardiopulmonary cerebral resuscitation (CPCR) room from 2016 to 2018. Patients aged over 18 years of age, with a diagnosis of lung contusion on either their early chest CT scan at arrival, or had diagnosed later on as a primary cause of lung contusion were included in the study.

Pulmonary contusion was evaluated by size and density according to the Hounsfield scale and the extent of involvement in each lung. Also, positive chest CT findings such as hemorrhage, pneumothorax, vascular disorders, diaphragmatic rupture, mediastinal injury, and rib fracture were evaluated and recorded simultaneously.

The patients were examined for symptoms of pulmonary contusion. They were then examined for any other nonthoracic injuries such as liver, spleen, and other visceral injuries in the abdomen. Factors such as the Glasgow Coma Scale (GCS) score, hospitalization duration, as well as the need and duration for ICU admission were also recorded.

The primary outcome of interest was hospital mortality. Secondary outcomes included pulmonary complications such as the development of hemothorax, admittance to the ICU and duration, and the need for intubation, and the possibility of tracheostomy requirement was evaluated and recorded.

Data were entered into SPSS software version 23, and statistical analysis was performed with the same software. Measurement data were described by mean ± standard deviation (SD), and numerical data were described by number (%). Statistical differences were assessed using Pearson's chi-square or Fisher's exact tests for categorical variables, and the paired-sample *t*-test was used to evaluate continuous variables, as appropriate. All analyses were performed in SPSS version 26.0, and *P* < 0.05 was considered statistically significant.

## 3. Results

In this study, 434 patients with the diagnosis of lung contusion were enrolled.  Table 1 demonstrates the clinical features and outcomes of these patients. The mean age of the participants was 41.17 (standard deviation: 17.89). Also, patients who were transferred to the ICU had an average length of stay of 7.69 days. The mortality rate in our study was 15.2% (66 patients).

Based on independent sample *t*-test analysis, the mean age of the patients who were deceased in our study was significantly higher than those who survived (54.56 ± 21.39 vs. 38.77 ± 16.01, respectively; *P* < 0.001)

Of the 281 patients who were reexamined for lung injury, 127 patients (33.4%) had an increase in severity or extent of lung contusion; however, this was not associated with the patient's mortality (*P*=0.057). Also, the rate of tracheostomy in patients with a pulmonary contusion was 16.7%.

Of the 170 patients with GCS of less than 8, 154 patients (74.4%) were intubated, and of the 264 patients with GCS greater than 8, 53 patients (25.6%) were intubated. There was also a significant association among GCS level and intubation of the patients (*P* < 0.001)

Of the patients who died in our study, 11 patients (21.6%) had an increase in severity or extent of lung injury in their re-examination; however, there was no significant association among progression of lung injury and mortality (*P*=0.057)

## 4. Discussion

Pulmonary contusion is the most common injury in blunt trauma. The prevalence of 25–75% is reported in different studies. The purpose of this study was to determine the clinical course of traumatic injuries with an initial diagnosis of a pulmonary contusion in patients referred to Shahid Rajaee Hospital CPCR from 2016 to 2018.

Pulmonary contusion patients usually sustain multiple injuries due to severe trauma mechanisms, in which other injuries may be life-threatening and more severe than respiratory symptoms, resulting in a delayed radiological evaluation of the pulmonary contusion. Therefore, monitoring and reassessment of traumatic patients are necessary for the diagnosis of pulmonary contusion [15].

In the majority of cases in our study, pulmonary contusions were associated with other injuries, most of which were head (56.9%) followed by limb (30%), pelvis (14.3%), abdomen (12.4%), and spine (9%) injuries. The results of this study, along with previous studies, emphasize the necessity of investigating and managing other injuries in lung injury patients as well as considering the possibility of lung contusion in traumatic patients with life-threatening injuries [16].

In general, various studies have reported an incidence of 25% to 35% of pulmonary blunt trauma, which accounts for more than 25% of blunt trauma mortality [17]. In our study, out of the 399 patients diagnosed with lung injury, 66 patients (15.2%) died. Of the patients who died due to lung injury, 11 patients (21.6%) had an increased severity or extent of lung injury in their re-examination. However, this was not significantly associated with their mortality.

In a retrospective study published in 1994, Kollmorgen stated that 70% of deaths from pulmonary congestion were due to lung injury or pulmonary failure [18]. ICU admission and mortality due to comorbid injuries have increased in various studies [6], which is consistent with the results of this study.

Of the 434 patients diagnosed with lung contusion who were included in the study, 206 patients (49.6%) were transferred to the ICU, with an average ICU stay of 7.69 days. In a peer-reviewed study in Canada, results showed that 84% of patients admitted to the ICU had a mean duration of 9.9 days [19]. Although the number of patients transferred to the ICU was higher in the present study, which may be due to the difference in the severity of trauma and vehicle safety in Iran and Canada, the length of stay in the ICU was approximately 2 days shorter, possibly due to better initial patient management in this center.

Evidence suggests that a flail chest is associated with a 75% higher risk of lung injury which can double the mortality rate [17,20,21]. Floating rib or thoracic fractures increase the risk of underlying tissue injury by up to 15%, although significant damage to lung tissue without chest fracture may also occur, and in our study, 45 (10.4%) had right-sided rib fracture and 47 (10.8%) had left-sided rib fracture [17]. Other studies have shown that lung injury associated with rib fractures is more localized and limited. A retrospective study of 100 patients also showed that lung injury in children is 18% less likely than that in adults to be associated with rib fractures [17].

Tyburski et al. found that up to 25% of patients diagnosed with lung injury developed with exacerbation on symptoms in the emergency within 24 hours of admission, despite the increased fluid levels in the lungs within 72 hours after trauma. Based on this study, most patients suffering from lung contusion trauma recovered within 7 to 14 days with minimal long-term effects [22]. Of the patients who were re-examined for lung injury, 127 patients (33.4%) had an increase in severity or extent of lung injury which confirms the results of the previous study.

A study by Richardson et al. reported successful management of patients with pulmonary contusion, in which 80% of them were managed without intubation and 50% of patients with flail chest [23]. It seems that, regardless of the mechanisms and severity of trauma and its variations in different countries, we should pursue an approach to reduce the rate of endotracheal intubation at these centers; however, it should be considered that patients with suspicion of respiratory failure should be intubated as soon as possible before exacerbation of symptoms [6].

In studies of blunt trauma patients, the rate of tracheostomy was not significantly different between patients with pulmonary contusion [23]. In this study, the rate of tracheostomy in patients with a pulmonary contusion was 16.7%, and it seems that tracheostomy has no special clinical significance in lung contusion patients compared to other patients.

Although we did not evaluate minimal invasive or laparotomy procedures, some studies suggest that the usage of laparoscopy in polytraumatic patients especially lung contusion may result in better respiratory function, faster recovery, fewer tracheostomies, and respiratory infections [24, 25]. Moreover, the use of emergency thoracotomy in blunt chest trauma is still a matter of debate, and several guidelines have been proposed to reduce the mortality and morbidity of patients considering the cost efficacy of the procedure [26].

There were some limitations in our study; generally, retrospective hospital surveys have been criticized for not providing precise estimates of disease incidence as not all hospitals in a particular region or district are included in the study. Also, this study was conducted based on hospitalized patients due to the unavailability of all records of lung contusion pattern data in the whole province. The second limitation was difficulty to precisely explain causal relationships due to the fact that the study was cross-sectional.

## 5. Conclusion

Contusion of lung parenchyma is a frequent result of severe blunt trauma to the thorax. Although recent advances in pulmonary care and ventilator management have been developed, there is still considerable morbidity and mortality associated with this condition. The severity and mechanisms of trauma and road safety vary in different countries; therefore, there is a need to provide a national guideline based on native patient information for better management.

## Figures and Tables

**Table 1:** Clinical features and outcomes of lung contusion patients in southern Iran.

Variable	Frequency (%) *n* = 434	Mortality (%)		*P* value ^*∗*^
		Yes *n* = 66	No *n* = 368	
Sex				
Male	366 (84.3)	57 (15.6)	309 (84)	0.716
Female	68 (15.7)	9 (13.6)	59 (16)	

Side				
Left	345 (79.5)	57 (86.4)	288 (78.3)	0.141
Right	349 (80.4)	53 (80.3)	296 (80.4)	1.000
Bilateral	260 (59.9)	44 (16.9)	216 (58.7)	0.275

Rib fracture				
Right	45 (10.4)	3 (4.5)	93.3 (11.4)	0.123
Left	47 (10.8)	7 (10.6)	40 (10.9)	1.000

Other location injuries				
Head and neck	247 (56.9)	47 (71.2)	200 (54.3)	0.015
Abdominal	54 (12.4)	11 (16.7)	43 (11.7)	0.309
Pelvic	62 (14.3)	13 (19.7)	49 (13.3)	0.182
Spinal cord	39 (9)	4 (6.1)	35 (9.5)	0.486
Limb	130 (30)	28 (42.4)	102 (27.7)	0.020

Lung injury				
Pneumothorax	90 (25.0)	16 (31.4)	79 (24)	0.297
Hemothorax	60 (15.8)	5 (9.8)	55 (16.7)	0.226
Both	32 (8.4)	4 (7.8)	28 (8.5)	1.000

GCS index				0.006
Equal or lower than 8	170 (39.2)	36 (54.5)	134 (36.4)	
Above 8	264 (60.8)	30 (45.5)	234 (63.6)	

Outcome				
Transfer to ICU	206 (49.6)	15 (24.2)	191 (54.1)	<0.001
Intubation	207 (47.7)	46 (69.7)	161 (43.8)	<0.001
Progression in follow-up CT scan	127 (33.4)	11 (21.6)	116 (35.3)	0.057

^*∗*^ Based on the chi-square test.

## Data Availability

SPSS data of the participant can be requested from the authors. The data used to support the findings of this study are available from the corresponding author upon request.
